# TREATMENT OF THORACOLUMBAR FRACTURES BY CLOSED REDUCTION VIA A PERCUTANEOUS SOLID PEDICLE SCREW

**DOI:** 10.1590/1413-785220233101e259041

**Published:** 2023-04-17

**Authors:** Changzhi Cheng, Guiqian Li, Yuanguo Luo, Zhoudan Lin

**Affiliations:** 1 923rd Hospital of People's Liberation Army, Department of Orthopedics, Nanning, Guangxi, China.

**Keywords:** Closed Fracture Reduction, Spinal Fractures, Pedicle Screws, Fracture Fixation, Internal, Redução Fechada, Fraturas da Coluna Vertebral, Parafusos Pediculares, Fixação Interna de Fraturas

## Abstract

**Objectives:**

Investigate the effect of closed reduction and per- cutaneous pedicle screw fixation in treating thoracolumbar fractures.

**Methods:**

This retrospective study analyzed 12 cases of single-segment thoracolumbar spine fractures without spinal cord and nerve injury at our department from March 2016 to September 2017. Patients were treated with closed reduction, percutaneous reduction, and internal fixation with solid pedicle screws. The operation time, intraoperative blood loss, anterior vertebral body height ratio (AVHR), Cobb angle (CA) of sagittal kyphosis, and VAS of back pain were determined and statistically compared.

**Results:**

The average operation time was 147.2 ± 45.6 min, and the average intraoperative bleeding was 67.8 ± 34.2 mL. All fractured vertebrae were completely reduced, their height was restored, and kyphosis was corrected. The average follow-up period was 10.6 ± 2.7 months, with significant improvements seen in the AVHR, CA of sagittal kyphosis, and VAS score (P < 0.01). One case had a broken rod after three months, and another had a postoperative infection. All the patients achieved bony healing.

**Conclusion:**

The treatment of thoracolumbar fractures by closed reduction and internal fixation with a percutaneous solid pedicle screw is simple, effective, and economical. ***Level of Evidence VI; Therapeutic Study, Case Series***.

## RESUMO

**Objetivo:**

Investigar o efeito da redução incruenta e da fixação com parafuso de pedículo percutâneo no tratamento das fraturas toraco- lombares.

**Métodos:**

Este estudo retrospectivo analisou 12 casos de fraturas toracolombares com segmento único sem lesão medular ou neural, encontrados no departamento dentro do período de março de 2016 a setembro de 2017. Os pacientes foram tratados com redução fechada e fixação interna com parafusos de pedículo percutâneo. O tempo de operação, a perda sanguínea intra-operatória, a relação da altura do corpo vertebral anterior (AVHR), o ângulo de Cobb (CA) da cifose sagital e a EVA relativa à dor nas costas foram determinados e comparados estatisticamente.

**Resultados:**

O tempo médio da operação foi de 147,2±45,6 min, com sangramento intraoperatório médio de 67,8±34,2 mL. Todas as vértebras fraturadas foram com- pletamente reduzidas, suas alturas foram restauradas e a cifose foi corrigida. O período médio de acompanhamento foi de 10,6±2,7 meses, apresentando melhorias significativas observadas no AVHR, CA da cifose sagital e pontuação VAS (P <0,01). Um caso teve uma haste quebrada após 3 meses, e outro paciente apresentou uma infecção pós-operatória. Todos os pacientes alcançaram a recuperação óssea.

**Conclusão:**

O tratamento das fraturas toracolombares por redução fechada com fixação interna pelo parafuso de pedículo percutâneo é simples, eficaz e econômico. ***Nível de Evidência IV; Estudos Terapêuticos, Série de Casos***

## INTRODUCTION

Recent developments in minimally invasive technology for spinal surgery include the continuous invention and improvement of minimally invasive surgical instruments. For thoracolumbar fractures without spinal cord and nerve injury, more spine surgeons are starting to use the minimally invasive method of closed reduction and percutaneous pedicle screw and screw rod system reduction and fixation, and its clinical applications are becoming more extensive as well.^1–3^ However, the operation method and instruments used still have shortcomings with regard to positioning, screw placement, and reduction, which to a certain extent affects the procedure and its outcomes. Fortunately, the invention and application of a new solid pedicle screw system was able to remedy this. Since March 2016, our department has used this procedure in some thoracolumbar fractures and has achieved good results.

## MATERIALS AND METHODS

This was a retrospective study based on medical records from the ar- chives of our Hospital from March 2016 to September 2017 (Table 1). This clinical study was approved by our Hospital Medical Ethical Committee (No 923ll-ky-2023-002-01), and informed consent for the surgical procedures and inclusion of data was obtained from all patients. A total of 8 males and 4 females with an average age of 34.8 years (range: 20–58 years) were enrolled in the study. There were 6 cases of L1 fracture, 3 cases of T12 fracture, 2 cases of L2 fracture, and 1 case of L5 fracture, all of which were single- segment fractures, comprised of 6 cases of falling injury, 4 cases of traffic accident injury, and 2 cases of heavy injury. All of them were AO type A fractures, with 5 cases of vertebral canal without space-occupying bone mass, and 7 cases with different degrees of fracture mass protruding into the vertebral canal. The affected volume was less than 1/3, and except in 1 case was more than 1/2. A total of 7 cases were operated within 72 h, 4 cases within 5 days, and 1 case within 9 days. The clinical manifestations of all patients were lumbago and back pain, limited lumbar motion, and no lower extremity sensory, motor, or fecal dysfunction. Before the operation, X-ray, CT, and MRI were performed in all cases. There was no absolute contraindication.

### Treatment method

#### Position reduction

All the patients were placed on a pad thin pillow immediately after admission to the hospital. Analgesics were given, and the thickness of the pillow was gradually increased if this was comfortable for the patient.

**Figure 1:**
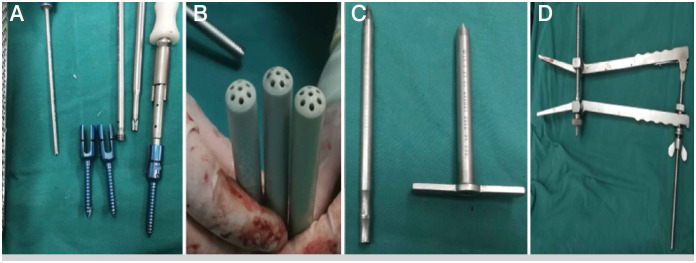
Percutaneous solid pedicle screw system: A, solid pedicle screw; B, positioning regulator; C, working channel; D, distractor.

After general anesthesia, the injured vertebra and the pedicles of the upper and lower vertebra were initially positioned via a positioner under G-arm fluoroscopy. After disinfecting and spreading the towel, the needle was inserted about 0.5–1 cm into the lateral projection of the pedicle to locate the positioning needle. If there is any deviation after fluoroscopy, the original needle can be retained. A special positioning regulator (Figure 1) was inserted through the original needle, and another needle was inserted into that positioning regulator in order to make the corresponding adjustment according to the deviation direction of the original needle. The positioning needle was directly adjusted by slowly hammering it into the vertebral body; fluoroscopy was performed during this time to ensure that the positioning needle was located in the pedicle, until the positioning needle was located in the middle of the vertebral body. A 2-cm longitudinal incision was made along the positioning needle, cutting the skin, subcutaneous tissue, and fascia, and performing blunt muscle separation. Then, the soft tissue expander was inserted along the positioning guide needle, leaving the outer sheath as the channel. Next, the opening of the bone opener was inserted, the bone was drilled, and tapping was performed. Finally, the positioning needle was pulled out, and the appropriate solid pedicle screw was inserted. In general, a single-side pedicle screw was placed in the injured vertebrae, and two pedicle screws were placed in the upper and lower vertebrae. After the screws were placed, a special small pull hook was placed on both sides of the screw to expose the U-shaped groove of the screws. After measurement, a titanium rod of appropriate length was pre-bent and then placed in the U-shaped groove. The operating table was adjusted to raise the head and tail of the bed. A special distractor was placed at the joint of the screw and the titanium rod. The lever principle was used to carry out the operation of vertebral body distraction and reduction. Fluoroscopy was used to confirm whether the reduction of the fracture vertebral body was satisfactory, the height of the vertebral body was restored, and kyphosis was corrected. Later, the nuts were tightened, the incision was closed, and drainage was placed.

**Table 1:** Patient demographic data.

**Demographic data Number**
Male gender 8
Female gender 4
Age, years (range) 34.8 (20–58)
**Mechanism of injury**
Fall from height 6
Road traffic accident 4
Heavy object injury 2
**Site of vertebral fracture (%)**
T12 3 (25.0)
L1 6 (50.0)
L2 2 (16.7)
L5 1 (8.3)

### Postoperative management

After the operation, the sensation and movement of the lower limbs were observed closely. Antibiotics were used to prevent infection. The drainage tube was left for 1–2 days, and it was removed after the drainage fluid was less than 50 mL. On the second day after the operation, the patients could get out of bed with braces, but bed rest was still advised. The brace was fixed for 3–4 months. After the operation, the anteroposterior (AP) and lateral view X-rays were reviewed regularly to observe the fracture healing, vertebral height, and physiological curvature of the spine.

### Statistical analysis

Data were recorded before and after the operation and at the last follow-up visit. The data included height of the vertebral body, Cobb angle of sagittal kyphosis, and visual analog scale (VAS) score; these were compared via the SPSS10.0 software(SPSS Inc., Chi- cago, IL) for the t-test, with P < 0.01 considered to be statistically significant.

## RESULTS

**Figure 2:**
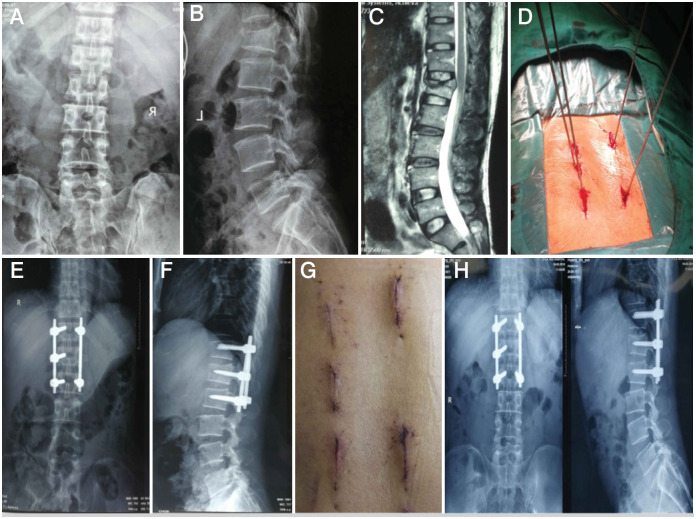
A 32-year-old man with a burst fracture of the lumbar vertebrae. A, B: preoperative X-ray; C: preoperative MRI; D: intraoperative positioning and then inserting the guide needle; E, F: postoperative X-ray; G: postoperative incision; H: review of X-ray after 1 year of operation.

The operation time lasted an average of 147.2 ± 45.6 min (range: 95–240 min), and the average intraoperative blood loss was 67.8 ± 34.2 mL (range: 20–120 mL). Postoperative follow-up happened after an average of 10.6 ± 2.7 months (range: 6–13 months). All fractured vertebrae were completely reduced, the height of the vertebrae was restored, and kyphosis was corrected. The height of the vertebral body, sagittal kyphotic Cobb angle, and VAS scores, taken before the operation, the second day after the operation, and at the last follow-up, are all shown in Figure 2 and Table 2. All these variables significantly improved after the operation and at the last follow-up (P < 0.01). After 3 months, 1 case had a broken rod, but the fracture healed well after follow-up examination, no screw loosening was found, and the patient had no uncomfortable symptoms. Another patient had postoperative infection, but after antibiotic treatment, symptoms improved and the patient was discharged from the hospital. Half a year later, the upper vertebra space collapsed, but this recovered to normal after a reoperation.

**Table 2:** Perioperative anterior vertebral body height ratio (VBH), Cobb angle of sagittal kyphosis (CA), and visual analog scale (VAS) (x ± s, n = 12).

	VBH CA	VAS
Preoperative	72.4 ± 9.4 14.4 ± 6.7	6.6 ± 0.9
Postoperative	99.5 ± 8.1* 6.5 ± 4.3*	1.9 ± 0.6*
Last follow-up	97.6 ± 8.4* 6.9 ± 4.3*	0.8 ± 0.7*

* Compared with postoperative, final follow-up, P < 0.01.

## DISCUSSION

Most thoracolumbar fractures can yield good results through con- servative treatment. With the invention and application of pedicle screws, surgical treatment has become an important means of thoracolumbar fracture treatment. It can restore the height of the vertebral body, correct kyphosis, immediately relieve symptoms, and get patients out of bed as soon as possible, greatly reducing the incidence of bed complications.

With the advancements of minimally invasive surgery, percutaneous pedicle screw placement technology has developed rapidly as well, which can improve the current surgical treatment for thoracolumbar fractures.^4–6^ Percutaneous pedicle screw insertion technology is usually operated step-by-step under the fluoroscope of a C-arm or G-arm with the help of special surgical instruments. Currently, the most commonly used percutaneous pedicle internal fixation systems are the Viper system from DePuy, the sextant system from Medtronic, the Matis system from Stryke, and the upass system from the domestic company Weigao; these are all hollow pedicle screw systems. The minimally invasive treatment of vertebral fractures has achieved good results and is already being widely used.^7,8^ However, there are still some weaknesses in the hollow pedicle system. First, there is no advantage in the positioning process. Although there is C-arm or G-arm fluoroscopy, it is not easy to find the ideal screw entry point accurately in the closed state, which is completely based on the experience and hand feeling of the operator. Second, most of the hollow screws are multiaxial screws, and the strength of their vertebral body reduction is not enough. Third, the design of reduction equipment is unreasonable and does not yield good results in terms of vertebral body reduction.

The key to reducing vertebral fractures lies in the reduction ability and maintenance ability of the screws. Arbash et al.^9^ found that, although there is no statistical difference between solid and hollow screws in maintaining the correction of kyphosis and vertebral height, the former has stronger correction ability. The reduction devices and techniques also play an important role. The improvement of several reduction devices and the reasonable application of reduction techniques have achieved satisfactory reduction results.^10–13^ In light of the previously mentioned problems, He Xinning^14^ designed a percutaneous solid pedicle screw system. This system uses a short tail solid uniaxial pedicle screw, with a so- phisticated positioning needle regulator and distraction reduction forceps, so that the whole operation process is easier and more programmed, and the effect is better. It has three advantages. First, as long as the first puncture of the positioning needle is not too biased, most of them can be corrected by inserting the positioning needle into its regulator and then inserting another positioning needle according to the direction of deviation; this saves time from the traditional method of positioning with a fluo- roscope. Second, the solid uniaxial pedicle screw has the same effect as a conventional open screw in its fixation and distraction functions, making it better than the hollow multiaxial screw. Third, the design of the distraction reduction forceps makes full use of the mechanical principle of the lever and combines it with the position reduction before and during the operation; thus, reduction of the vertebral body becomes very easy.

In our study, the percutaneous solid pedicle screw system was used for minimally invasive surgery, and the whole process of positioning and puncture was relatively smooth. Compared with the previous hollow screw fixation system, the positioning time was greatly shortened, and the number of fluoroscopy was significantly reduced, which was greatly beneficial for patients and doctors. Although it is a solid screw, the process of pedicle screw placement is not different from that of a hollow screw. Depending on the preparation of a good working channel, screw placement is generally easy. The greatest advantage of this set of solid screws lies in its strong ability to reduce vertebral fracture. The unique fixation characteristics of uniaxial screws, which have more strength to open and reduce the vertebral fractures, make up for the disadvantages of multiaxial screws with uncertain direction. In combination with the role of body position reduction and distractor, most of the fractured vertebral bodies were completely reduced, and the height of the vertebral body was restored, kyphosis was corrected, and the bone blocks protruding into the vertebral canal were restored. No case needed open reduction or decompression.

The study showed that, except for a few cases with a long operation time in the early stage, the operation time in the later stage was significantly shortened due to the improvement of the proficiency of instrument operation. Generally, the operation can be completed in over an hour, and the amount of blood loss in the operation was very small, with an average of about 40 mL. The postoperative pain was significantly improved, and the VAS score significantly improved after the operation and the last follow-up. The height of the vertebral body generally recovered after the operation. Although some cases lost height at different degrees at the last follow-up, this had no significant difference compared with the postoperative data. The Cobb angle of sagittal kyphosis decreased after the operation, and this was slightly lost in some cases at the last follow-up, but this was not significantly different compared with the postoperative data. We also analyzed reasons for the loss of the height of the vertebral body and Cobb angle of the sagittal kyphosis of the spine during the last follow-up. Two main factors are considered. The first is being allowed to leave bed too early. Although the fractured vertebral body can be reduced by surgery, it is difficult for the cancellous bone to reexpand, leaving a gap in the vertebral body. If the patients start to walk without complete bone healing, it may be difficult to maintain the dimension only through the support of the screws, so the height of the vertebral body is lost and it is easy to break the screw and the rod. In our group, there was a broken rod. Although the final fracture healing was good, it also increased the risk of fracture healing and reduced the quality of fracture healing. The second factor is that there is no bone graft in the vertebral body and the space in the vertebral body is not filled, affecting the speed of fracture healing, thus leading to the collapse of the vertebral body after getting out of bed. On the basis of the above, there are two areas of improvement. The first is to carry out intravertebral bone grafting and transpedicular allogeneic bone grafting. The second is to either appropriately delay the time of getting out of bed or impose restrictions when doing so. This can be carried out by wearing supportive protection and strictly controlling the time spent outside of bed. A number of studies have confirmed that percutaneous pedicle screw reduction and fixation at the fractured vertebra for thoracolumbar fractures is associated with better recovery and a maintained height of the injured vertebra.^15,16^ In the later cases of our group, the method of fixation and reduction of the injured vertebra by a percutaneous pedicle screw at the fractured vertebra was used routinely with positive outcomes.

Although the percutaneous solid pedicle screw system has obvious advantages for the reduction of thoracolumbar fractures, there are still some problems in the system that need further improvement. For example, the screw can be designed as a long tail nail to make titanium rod implantation and other operations easier. Some improvements of the distractor also need to be made in order to make the procedure easier.

## CONCLUSIONS

In this preliminary retrospective study of 12 patients, we have shown that closed reduction and percutaneous internal fixation using a solid pedicle screw system in the treatment of thoracolumbar fractures could effectively restore and maintain vertebral height. This is a relatively simple, acceptable, low-cost, and minimally invasive surgical choice for patients with type A thoracolumbar fractures.

The study was conducted at the 923rd Hospital of People's Liberation Army, Nanning, Guangxi, China.
